# The cost-effectiveness of digital breast tomosynthesis in a population breast cancer screening program

**DOI:** 10.1007/s00330-020-06812-x

**Published:** 2020-05-07

**Authors:** Jing Wang, Xuan-Anh Phi, Marcel J. W. Greuter, Alicja M. Daszczuk, Talitha L. Feenstra, Ruud M. Pijnappel, Karin M. Vermeulen, Nico Buls, Nehmat Houssami, Wenli Lu, Geertruida H. de Bock

**Affiliations:** 1 Department of Epidemiology, University of Groningen, University Medical Center Groningen, Groningen, The Netherlands; 2 Department of Radiology, University of Groningen, University Medical Center Groningen, Groningen, The Netherlands; 3 Department of Radiology, Vrije Universiteit Brussel, Universitair Ziekenhuis Brussel, Brussels, Belgium; 4 Department of Radiology, University Medical Center Utrecht, Utrecht University, Utrecht, The Netherlands; 5 Sydney School of Public Health, Faculty of Medicine and Health, University of Sydney, Sydney, Australia; 6 Department of Epidemiology and Health Statistics, Tianjin Medical University, Tianjin, China

**Keywords:** Breast neoplasms, Mammography, Tomosynthesis, Mass screening, Cost-benefit analysis

## Abstract

**Objectives:**

To evaluate at which sensitivity digital breast tomosynthesis (DBT) would become cost-effective compared to digital mammography (DM) in a population breast cancer screening program, given a constant estimate of specificity.

**Methods:**

In a microsimulation model, the cost-effectiveness of biennial screening for women aged 50–75 was simulated for three scenarios: DBT for women with dense breasts and DM for women with fatty breasts (scenario 1), DBT for the whole population (scenario 2) or maintaining DM screening (reference). For DM, sensitivity was varied depending on breast density from 65 to 87%, and for DBT from 65 to 100%. The specificity was set at 96.5% for both DM and DBT. Direct medical costs were considered, including screening, biopsy and treatment costs. Scenarios were considered to be cost-effective if the incremental cost-effectiveness ratio (ICER) was below €20,000 per life year gain (LYG).

**Results:**

For both scenarios, the ICER was more favourable at increasing DBT sensitivity. Compared with DM screening, 0.8–10.2% more LYGs were found when DBT sensitivity was at least 75% for scenario 1, and 4.7–18.7% when DBT sensitivity was at least 80% for scenario 2. At €96 per DBT, scenario 1 was cost-effective at a DBT sensitivity of at least 90%, and at least 95% for scenario 2. At €80 per DBT, these values decreased to 80% and 90%, respectively.

**Conclusion:**

DBT is more likely to be a cost-effective alternative to mammography in women with dense breasts. Whether DBT could be cost-effective in a general population highly depends on DBT costs.

**Key Points:**

*• DBT could be a cost-effective screening modality for women with dense breasts when its sensitivity is at least 90% at a maximum cost per screen of €96.*

*• DBT has the potential to be cost-effective for screening all women when sensitivity is at least 90% at a maximum cost per screen of €80.*

*• Whether DBT could be used as an alternative to mammography for screening all women is highly dependent on the cost of DBT per screen.*

**Electronic supplementary material:**

The online version of this article (10.1007/s00330-020-06812-x) contains supplementary material, which is available to authorized users.

## Introduction

Breast cancer is one of the leading causes of death among women, and the most common cancer in women—approximately 1 in 8 women will develop breast cancer in their lifetime [1]. National mammography screening programs have been introduced in most European and developed countries for several decades. The key benefit of regular mammography screening is that it detects breast cancer at an early stage allowing more effective treatment and improved survival [2]. A recent study showed that the incidence of early-stage breast cancer in patients who attend screening regularly is significantly higher than that in patients who did not, suggesting a stage shift to earlier detection with the implementation of regular screening [3]. However, there are also controversies regarding screening. Over-diagnosis and related over-treatment are considered a main drawback of screening, with estimates of over-diagnosis varying from 15 to 30% [4]. In addition, false-positive results lead to unnecessary biopsies and negative psychological outcomes which can decrease the efficacy and acceptability of screening programs [4]. A key issue is the imperfect sensitivity of digital mammography (DM) which reduces the effectiveness of screening [5].

It is well-documented that mammographic sensitivity decreases with increasing breast density [6]. Moreover, a higher breast density is an additional risk factor for breast cancer, and both false-positive and false-negative interpretations are more likely with dense breasts [7]. Nowadays, digital breast tomosynthesis (DBT) is gaining widespread attention because it improves the detection of cancer particularly in dense breast tissue. When using adjunct DBT in women with DM-negative dense breasts, the ASTOUND-2 trial showed an incremental cancer detection rate (CDR) of 2.83 per 1000 screens [8]. In population-based screening, a recent meta-analysis showed that DBT combined with DM yields a pooled incremental CDR of 2.4 cancers per 1000 screens in biennial screening practice with only a slight increase in recall rate compared to DM alone [9]. With respect to the cost-effectiveness of DBT, the evidence is limited. However, in a study focusing on US population aged 50–74 years, biennial combined DM and DBT screening for women with dense breasts was reported to be cost-effective if the price of DBT plus DM was below $226 [10]. Recently, synthesised 2D mammograms from DBT were introduced showing similar sensitivity and specificity with respect to DM [11]. This has made it feasible to use DBT-only acquisition as a stand-alone screening modality instead of combined DBT and DM [12].

In this evolving landscape for population breast screening, we undertook a study aiming to evaluate at what sensitivity DBT could be a cost-effective alternative to DM in a population breast cancer screening program, while the specificity is kept constant. A validated micro-simulation model (SiMRiSc) was applied to simulate biennial population breast cancer screening in The Netherlands for women 50–75 years of age, whereby DBT was applied to replace DM for all women or, alternatively, for women with high breast density, against DM screening as currently practiced. In doing so, we anticipate that the findings from this study could provide health-economic evidence to the Dutch program and many organized population screening programs practicing biennial DM screening. However, we need to address other important issues beyond health economics such as risk stratification and estimates of incremental mortality impact, and recall rate should also be taken into consideration before implementing DBT in a population screening program [13].

## Material and methods

This study was reported according to the Consolidated Health Economic Evaluation Reporting Standards (CHEERS) statement [14]. The simulation model on radiation risk and breast cancer screening (SiMRiSc) was applied in the analysis [15–17].

### Description of the model and input variables

SiMRiSc is a micro-simulation Markov model that was previously published and externally validated in the general population and in women with BRCA gene mutations. In summary, women’s lifetimes were simulated considering their life expectancy, chance of developing cancer, tumour growth, probability of tumour self-detection and survival probability from breast cancer. If a tumour was present at the screening moment, the chance of detection was determined by the mammographic sensitivity. The sensitivity is a function of breast density which depends on the age of the woman. After breast cancer diagnosis, either by screening or by self-detection, the woman was removed from simulation and the breast cancer age-specific death of the woman was calculated based on life expectancy after diagnosis which depended on tumour size. Also included were mammographic specificity for the introduction of false positives, and the probability of tumour induction due to the ionising radiation from mammography. In the model, we assumed an 80% participation rate and only invasive cancers were considered [18].

The estimates for the model variables were based on published data for population statistics from The Netherlands and the USA, results of systematic searches and published cost estimates (Table 1) [15–27]. The population statistics used in our study included cumulative lifetime risk of breast cancer, mean onset age of breast cancer, breast density distribution and participation rate, which are summarised in Table 1. In this study, all sensitivities and specificities are given as modality sensitivity and modality specificity respectively [28]. Because DBT is a relatively new technique, there are no published data on either breast density averaged sensitivity or sensitivities based on breast density from a screening setting with long follow-up. Therefore, we used a DBT sensitivity that was constant across breast densities, and varied the sensitivity values from 65 to 100% in steps of 5%, with a lower boundary set at the minimum estimate for DM sensitivity [25]. The DBT specificity was fixed at 96.5%, which was the same as the specificity of DM [24]. The cost of mammography in the Dutch national screening program was determined as the cost of the whole program divided by the number of participants [18]. The base price for DBT was estimated at 1.5 times the DM costs, i.e. €96 per screen, allowing for a conventional cost-effectiveness estimate, given the higher equipment costs, added digital storage capacity, more expensive reading stations and the double-reading time for DBT [29]. Additionally, a lower estimated cost for DBT was also simulated, which was 1.25 times the DM cost, i.e. €80 per screen. A detailed description of other input variables can be found in our previous studies [15–17].

**Table 1:** Input variables and their estimates for the SiMRiSc model

	Variable		Mean estimate (SD)				Reference
Population	Cumulative lifetime breast cancer risk at the age of 70		22.6% (0.74)				[19]
	Mean onset age of breast cancer		72.9 (1.1)				
	SD in onset age of breast cancer		21.1 (0.93)				
	Breast density distribution	Age group	BI-RADS density				[20–22]
			1	2	3	4	
		< 40	5%	30%	48%	17%	
		40–50	6%	34%	47%	13%	
		50–60	8%	50%	37%	5%	
		60–70	15%	53%	29%	3%	
		> 70	18%	54%	26%	2%	
	Participation rate		80%				[18]
Tumour induction model	Excess relative risk of tumour induction due to radiation per Gy		0.51 (0.16)				[17]
Tumour growth model	Tumour doubling time per age group	< 50	80 (28) days				[23]
		50–70	157 (25) days				
		> 70	188 (52) days				
Digital mammography	Sensitivity		BI-RADS density				[15]
			1	2	3	4	
			87%	84%	73%	65%	
	Specificity		96.5%				[24]
	Cost/screen		€64				[25]
	Mean glandular dose		3.0 (1.0) mGy				[17]
	Detection threshold		5 mm				[26]
Digital breast tomosynthesis	Sensitivity*		65–100%				[25]
	Specificity		96.5%				[24]
	Costs/screen		€96 /€80				
	Mean glandular dose		4.0 (1.3) mGy				[26]
	Detection threshold		5 mm				[17]
Costs in case of positive finding	Biopsy		€176				[27]
	Treatment (tumour diameter)	< 2 cm	€6438				[16]
		2–5 cm	€7128				
		> 5 cm	€7701				

*For sensitivity of DBT, the lower boundary was set at the minimum estimate for the DM sensitivity

*SD* = standard deviation

### Screening scenarios

Three screening scenarios were evaluated. In the first scenario, DBT was used for biennial breast cancer screening only for women with high-density breasts (BI-RADS 4th edition density scores 3 and 4), whereas DM was used for women with non-dense breasts (scenario 1). The breast density distribution was described as a percentage, according to age group, as defined in Table 1. In the second scenario, DBT was used for biennial breast cancer screening for all women aged 50–75 (scenario 2). The current breast cancer screening program in The Netherlands, biennial mammography screening with DM for women aged 50***–***75 years, was simulated as a reference scenario. To make appropriate comparisons among scenarios, we used the same input variables of population statistics, tumour induction model and tumour growth model***;*** however, the modality-related variables such as cost, dose and sensitivity were varied according to the specific modality used.

### Outcome of the simulation model

One hundred thousand women were simulated for all three scenarios in order to minimise the statistical error and keep the computation time within limits (approximately 0.15 min for each simulation on a PC workstation). Each simulation was repeated 10 times in order to calculate the error of the point estimates. The sensitivity of DBT was varied between 65 and 100% in steps of 5%. The results from the simulations were reported in terms of the number of screen-detected tumours, the number of interval cancers and the number of life years gained (LYG) for both DBT screening scenarios as compared to the reference. All results and the standard errors (SEs) were calculated based on the outcomes of the simulation repetitions.

### Cost-effectiveness analysis

Only direct medical costs were considered in this study, including the screening costs for different modalities, the treatment costs based on the tumour size and related biopsy costs due to positive screening results. Incremental cost-effectiveness ratios (ICER) for both DBT screening scenarios were estimated as the ratio of the additional costs to the additional LYG compared to the reference, and ICER was also estimated between the two DBT scenarios. The ICER threshold was set to €20,000 per LYG [30]. Discounting is the process of converting future costs to their present value; to reflect the fact that, in general, society prefers to receive benefits sooner rather than later, and pays costs later rather than sooner [31]. A discount rate of 3% for both costs and effects was applied allowing for international comparisons [32]. Additionally, a discount rate of 4% for costs and 1.5% for health effects (LYG) was applied according to the Dutch guidelines [33]. In the main text, all ICERs are reported as international discounted ICERs; the undiscounted and Dutch discounted ICERs are provided in the supplementary file.

## Results

The reference scenario of DM screening resulted in a cumulative count of 536 screen-detected cancers, 272 interval cancers and 1353 LYG when 10,000 women were screened biennially from age 50 to 74 years. The results based on modelled sensitivity of DBT showed that the effectiveness of both DBT scenarios increased at increased DBT sensitivity (Table 2) as more screen-detected tumours, fewer interval tumours and more LYG were observed. When the sensitivity of DBT was lower than 72%, both DBT scenarios were less effective than the reference scenario. Generally, compared to the reference (DM), the ICER for both DBT scenarios became more favourable with increasing DBT sensitivity.

**Table 2:** Screening outcomes of using biennial DBT in a population screening program

Sensitivity of DBT	65%	70%	75%	80%	85%	90%	95%	100%
DBT for dense breasts compared to DM (scenario 1–reference)								
*N* screen-detected	− 12 (0)	− 3 (0)	*+ 6 (0)*	*+ 15 (0)*	*+ 24 (1)*	*+ 31 (1)*	*+ 40 (1)*	*+ 47 (1)*
*N* interval	+ 12 (0)	+ 4 (0)	*− 4 (0)*	*− 12 (0)*	*− 19 (0)*	*− 25 (0)*	*− 32 (1)*	*− 39 (1)*
LYG	− 39 (2)	− 14 (2)	*+ 12 (2)*	*+ 40(4)*	*+ 63 (4)*	*+ 88 (5)*	*+ 115(5)*	*+ 138(5)*
Discounted^a^ LYG	− 14 (1)	− 5 (1)	*+ 5(1)*	*+ 19 (2)*	*+ 30 (2)*	*+ 42 (2)*	*+ 54 (2)*	*+ 65 (2)*
Discounted^b^ LYG	− 23 (1)	− 8 (1)	*+ 8 (2)*	*+ 27 (3)*	*+ 43 (3)*	*+ 60 (3)*	*+ 78 (3)*	*+ 94 (3)*
DBT for all women compared to DM (scenario 2–reference)								
*N* screen-detected	− 74 (1)	− 47 (1)	− 22 (1)	*+ 2(1)*	*+ 26(1)*	*+ 48 (0)*	*+ 71 (0)*	*+ 91 (1)*
*N* interval	+ 66 (1)	+ 43 (1)	+ 21 (0)	+ 1(1)	*− 19 (0)*	*− 38 (1)*	*− 57 (0)*	*− 74 (1)*
LYG	− 219 (3)	− 146 (4)	− 76 (3)	− 5 (4)	*+ 64 (4)*	*+ 129 (3)*	*+ 195 (4)*	*+ 254 (5)*
Discounted^a^ LYG	− 76 (1)	− 51 (1)	− 25 (1)	*+ 3 (2)*	*+ 31 (2)*	*+ 62 (3)*	*+ 92 (2)*	*+ 120 (3)*
Discounted^b^ LYG	− 126(2)	− 84(2)	− 43(2)	*+ 1 (3)*	*+ 44 (3)*	*+ 88 (4)*	*+ 133 (3)*	*+ 172 (4)*
DBT for all women compared to DBT for dense breasts (scenario 2–scenario 1)								
*N* screen-detected	− 62(1)	− 44(1)	− 28(1)	− 13(0)	*+ 3(0)*	*+ 17(1)*	*+ 31(1)*	*+ 44(1)*
*N* interval	+ 54(1)	+ 39(0)	+ 25(1)	+ 12(0)	0(0)	*− 12(0)*	*− 24(1)*	*− 35(1)*
LYG	− 180(3)	− 132(4)	− 88(3)	− 45(2)	*+ 1(1)*	*+ 41(2)*	*+ 80(3)*	*+ 117(3)*
Discounted^a^ LYG	− 62(1)	−46(1)	− 30(1)	− 16(1)	*+ 1(0)*	*+ 19(1)*	*+ 38(1)*	*+ 54(1)*
Discounted^b^ LYG	− 104(2)	− 76(2)	− 51(2)	− 26(1)	*+ 1(1)*	*+ 28(1)*	*+ 54(2)*	*+ 79(2)*

Data shown as differences in number of DBT screen-detected tumours (*N* screen-detected) and number of interval tumours (*N* interval) for scenarios 1 and 2 with respect to the reference scenario of biennial DM screening for women 50–75 years of age, and for scenario 2 with respect to scenario 1. All data expressed as mean (SEs) per 10,000 women screened. Discounting^a^: 3% for both cost and LYG. Discounting^b^: 4% for cost and 1.5% for LYG

*N* = number; *LYG* = life years gained; *DM* = digital mammography; *DBT* = digital breast tomosynthesis.

Values in *italics* indicate the scenario ourperforms the comparison in the screening outcome

### Scenario 1: DBT screening only for women with dense breasts

**Fig. 1:**
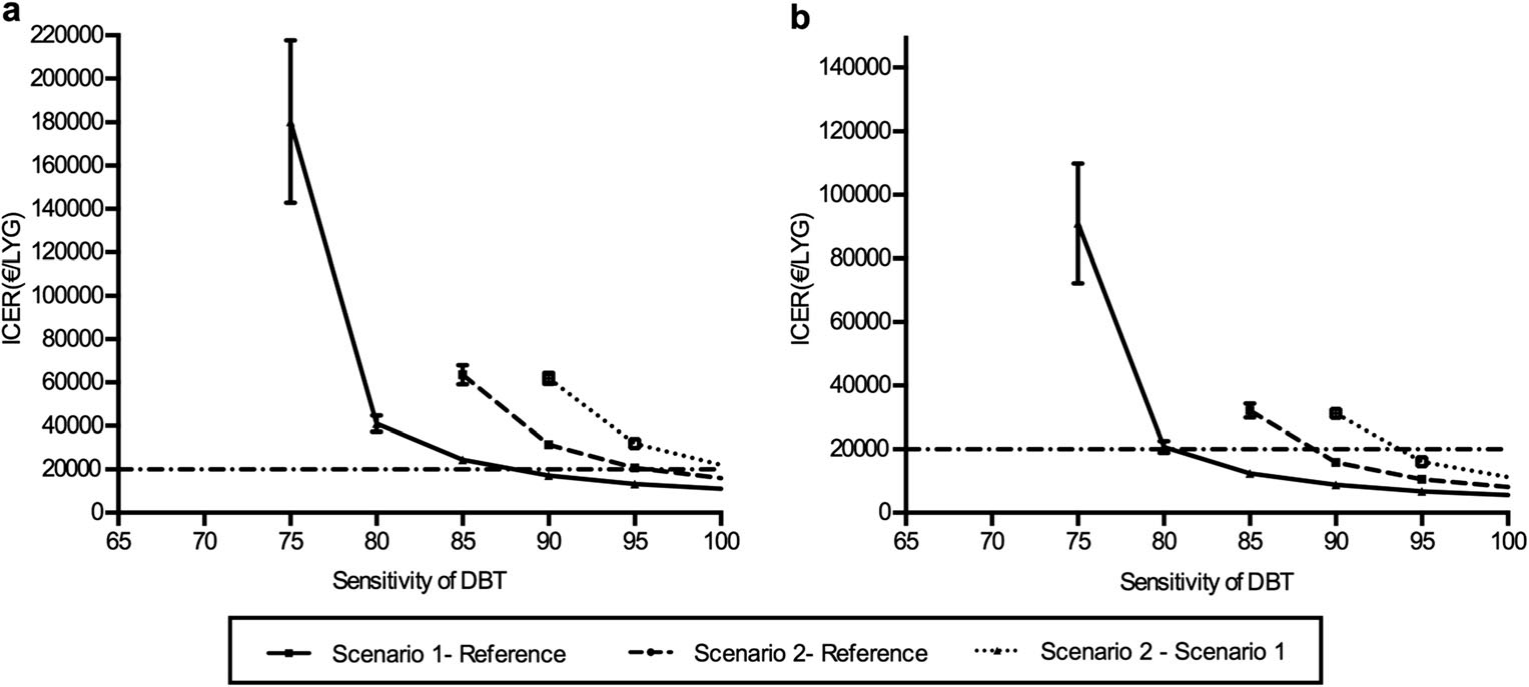
The discounted incremental cost-effectiveness ratio (ICER) as a function of the sensitivity of digital breast tomosynthesis (DBT) at a DBT cost of €96 (**a**), or at a DBT cost of €80 (**b**). Discount rate of 3% was applied to costs and life years gained. Scenario 1: DBT for women with dense breast; scenario 2: DBT for whole population; reference: DM for whole population. Abbreviations: ICER = incremental cost-effectiveness ratio

DBT screening only for women with dense breasts was the most effective scenario when the sensitivity of DBT was at least 72% (Table 2). Compared with the reference, when a 3% discounting rate was applied, at a cost of €96, scenario 1 was cost-effective at a sensitivity of approximately 86% (Table 3 and Fig. 1a), whereas at a cost of €80, scenario 1 became cost-effective when DBT sensitivity was at least 80%. (Table 3 and Fig. 1b).

**Table 3:** The cost-effectiveness of biennial screening using DBT in a population screening program

Sensitivity of DBT (%)	At €96 per DBT			At €80 per DBT		
	Scenario 1 compared to reference	Scenario 2 compared to reference	Scenario2 compared to scenario 1	Scenario 1 compared to reference	Scenario 2 compared to reference	Scenario2 compared to scenario 1
65	–	–	–	–	–	–
70	–	–	–	–	–	–
75	180.3 (37.4)	–	–	91.1 (18.9)	–	–
80	41.0 (3.8)	–	–	20.8 (1.9)	–	–
85	24.4 (1.6)	63.7 (4.5)	–	12.4 (0.8)	32.2 (2.3)	–
90	17.3 (0.8)	31.3 (1.3)	61.9 (2.6)	8.8 (0.4)	15.9 (0.7)	31.3 (1.3)
95	13.2 (0.5)	20.8 (0.5)	31.9 (0.9)	6.7 (0.2)	10.6 (0.3)	16.2 (0.5)
100	11.0 (0.3)	16.0 (0.4)	22.1 (0.6)	5.6 (0.2)	8.2 (0.2)	11.2 (0.3)

Data are reported as incremental cost-effectiveness ratios (ICERs) with associated standard errors. ICERs are expressed as 1000 euros per LYG. A discount rate of 3% was applied to costs and life years gained. Data which not shown in the table indicates that the specific scenario was dominated by the comparator

### Scenario 2: DBT for whole population

At a DBT sensitivity of 80%, the effectiveness of screening with DBT was comparable to screening with DM regarding the number of screen-detected cancers, interval cancers and LYGs (Table 2). When the sensitivity of DBT was > 85%, scenario 2 was the most effective resulting in at least 26 more screen-detected cancers, 19 fewer interval cancers and 64 more undiscounted LYG per 10,000 women than biennial DM screening (Table 2). However, at a cost of €96, scenario 2 would not be cost-effective unless the sensitivity of DBT was larger than 95% when a 3% discounting rate was applied (Fig. 1a). However, if the cost of DBT decreased to €80, scenario 2 could be cost-effective when DBT sensitivity was around 90% (Fig. 1b).

### Scenario 2 compared with scenario 1

When DBT sensitivity was lower than 85%, scenario 2 was always dominated by scenario 1. At a cost of €96, scenario 2 could never be cost-effective even the sensitivity of DBT was 100% when discounted by international rate (Fig. 1a), whereas scenario 2 could be cost-effective than scenario 1 w hen the sensitivity of DBT was around 95% at a cost of €80(Fig. 1b).

## Discussion

Our simulation modelling showed that for women aged 50–75 years, biennial screening with DBT for women with dense breasts is cost-effective when the sensitivity is around 90% (depending on the international discount rate), whereas DBT is unlikely to be a cost-effective alternative to DM if used to screen the general population when the cost of DBT is €96. However, if the price of DBT decreased to €80, the sensitivities to be cost-effective would decrease to 80% and 90% for scenarios 1 and 2, respectively. Our findings may be of immediate relevance to policy makers considering population breast cancer screening programs with DBT, or those planning density-tailored screening scenarios.

Instead of using a point estimate for DBT sensitivity, we used a sensitivity range to estimate the lowest sensitivity at which DBT would be cost-effective in a population breast cancer screening program, given the range of published DBT sensitivities. In population-based screening programs, the performance of DBT showed a similar or slightly higher sensitivity compared to DM, and the DBT sensitivity was varied between 81.1 and 91.6% [12, 34–36]. A recent meta-analysis restricted to women with dense breasts showed that the sensitivity of DBT or DBT plus DM was higher (84–90%) than DM alone (69–86%) [37]. Given the range of sensitivity values, the required threshold sensitivity in our findings suggests that only when the cost of DBT was around €80, DBT could be a cost-effective alternative to DM when applied for screening the whole population at a sensitivity of 90%. However, no matter which value of these two DBT costs is used, DBT screening only for women with dense breasts could be cost-effective, since the threshold for dense breasts is within the reported DBT sensitivity range. Another important issue is that previous studies have shown that DBT detects more tumours than DM [36, 38]. Therefore, the sensitivity of DM is likely to be overestimated when cancers that are only detected by DBT are not taken into account (unless there has been adequate follow-up). However, in a recent study using DM and DBT in over 24,000 women where DBT-only cancers were also counted, the modality sensitivity of DM was approximately 80% which is comparable to the overall modality sensitivity of 79% used in our study [36]. Nevertheless, as it is likely that there might still be some overestimation of DM sensitivity, this might lead to a slight underestimation of DBT cost-effectiveness. We expect, however, that this would not influence the main outcomes of our study.

DBT for population screening is becoming more widespread in some settings, based on cancer detection and recall metrics; however, there is limited evidence on whether DBT could be a cost-effective alternative to DM in a population screening program, and little knowledge exists on its long-term outcomes. Only two studies have so far compared the cost-effectiveness of DBT plus DM to DM for breast cancer screening, both done in the USA [10, 39]. Lee et al simulated biennial DBT plus DM among women aged 50–74 years with dense breasts using a discrete-event model, and the estimated sensitivity of DBT plus DM was 80%, which was lower than our estimated threshold [10]. The ICER was $53,893 which was considered to be cost-effective using a US threshold of $100,000 per QALY [40]. In our study, using a DBT sensitivity of 80%, at the cost of €96, the ICER for women with dense breasts was €41,021per LYG, generally similar results to Lee et al [10]. Kalra et al simulated annual DBT plus DM among women aged 40 years and older using a Markov cohort decision-analytic model [39]. The ICER was $20,230 per QALY, which was much lower than the ICER of screening the whole population as shown in our study.

The cost-effectiveness of DBT screening is highly dependent on the cost of DBT per examination. There are many factors that can influence DBT costs. Reading time is the main indirect cost related to DBT screening. Reading DBT images roughly doubles the interpretation time for mammograms [29]. Secondly, the price of new digital mammographic units with DBT capability entails an upfront investment [41]. A dedicated workstation is also needed to interpret DBT images, also requiring additional funding [41]. Information technology and image archiving infrastructure is substantially higher for DBT, for example DBT storage space can be 100 to 200 times that of DM [41]. In previous studies from the USA, the costs of adjunct DBT are found to be around 30–40% higher compared to DM alone [10, 39], while the To-Be trial in Norway reported an incremental screening cost of €8.5 per screen [42]. However, the cost estimated in the To-Be trial might be underestimated. Firstly, the screening machines ran at a 100% capacity throughout the study period, which might not always be the case in practice. Secondly, the cost per screen depends on the number of women screened, and the trial was conducted in a city with a large population density, the cost might be higher when generalised to a population program. Therefore, in our study, we used two estimated values, a conventional estimate for the price of DBT at 50% higher than DM, and a 25% higher estimate based on expert opinion.

Regarding specificity, there is no consensus on whether DBT specificity is improved compared to DM. Some studies showed that specificity can be improved with adjunct DBT compared to DM alone [43, 44]. However, a meta-analysis addressed that the recall rate of adjunct DBT increased slightly in European countries, where the recall rate of DM was relatively low compared to other regions such as the USA [9]. In addition, the Malmö screening trial, a prospective study conducted in Sweden, reported an increase in false positive recall rate for one-view DBT compared with two-view DM (1.7% vs 0.9%, respectively) [12]. In a recent meta-analysis restricted to women with dense breasts, the improvement in specificity from DBT was found to be inconsistent in a screening setting [37]. Because of these inconsistent results, the specificity of DBT in our analysis was fixed at a conventional estimate value of 96.5%, which was the same as the specificity of DM.

Our study used a validated model for which a sensitivity analysis has been done in a previous study indicating that the outcomes of the model were most sensitive to changes in the lifetime risk of breast cancer [17]. In our model, the sensitivity of DM depends on breast density and age, so increasing age generally leads to a decrease in breast density and to a corresponding increase in sensitivity of DM. This is an important component to consider as approximately 36% of Dutch women have dense breasts [45]. In addition, the model includes an increased risk of cancer induction due to radiation dose, which was not considered in previous studies that investigated the cost-effectiveness of DBT as a screening modality [10, 39]. Although we used a relatively higher dose for DBT compared to DM (4 mGy vs 3 mGy, respectively), only 0.4% more tumours were introduced by the increased dose. Therefore, we estimate that the influence of the increased dose from DBT has a negligible influence on the outcomes of our model.

As to limitations of the study, we point out that ductal carcinoma in situ is not included in this model. DCIS accounts for nearly 20% of the DM screen-detected tumours in the Dutch population and similar proportions are detected in other population screening programs [18, 46]. However, previous studies have shown that DBT plus DM can increase invasive CDR without preferentially increasing the proportion of DCIS compared to DM alone [47]. So, we expect that addition of DCIS into the model would not substantially change the major outcomes of the analysis. Another potential limitation is that we used values for DM sensitivity generated using meta-analysis [15]; this included some studies that were based on single-reading screening setting, suggesting that DM sensitivities might be lower compared to double-reading screening setting. The cost-effectiveness of DBT would be overestimated especially for scenario 2, where DBT was used for the whole population. Thirdly, it has been shown that specificity increases with a decrease in breast density [22]. There is, however, no reliable data on the dependence of specificity on breast density for DBT. Therefore, as a first-order estimation, we used a constant specificity which was independent of breast density in our model. Nevertheless, if a similar decrease in specificity for both DBT and DM was applied in dense breasts, the ICER in our study would not be influenced as the number of false positives would be similar for both DBT and DM, making the incremental cost nearly unchanged. In addition, when implementing scenario 1 in practice, it is likely that every woman would get a DBT examination in the first screening round in order to assess breast density from the synthetic mammogram. This results in a 1.1% increase in total costs. Therefore, in order for scenario 1 to be cost-effective, the sensitivity of DBT increases to 92% at a cost of €96 and approximately 85% at a cost of €80 per screen. Finally, we modelled the modality sensitivity of DBT, where we evaluated the effectiveness of a screening program. Because DBT is a relatively new technique, there are no data available on the sensitivity of a screening program including DBT, as long follow-up is needed to obtain reliable estimates of program sensitivity [28].

## Conclusion

Several European countries, and Australia, have recently evaluated DBT in trials performed in population-based screening programs [12, 35, 36, 48]. However, DBT is a relatively new technology, and whether DBT could be an alternative cost-effective replacement for DM in a population screening program is a matter of ongoing investigations. Based on our analysis, DBT is more likely to be a cost-effective alternative to DM in women with dense breasts. The threshold sensitivity for DBT to be cost-effective is 90% and 80% at €96 and €80, respectively. Whether DBT could be cost-effective in a general population highly depends on the DBT cost. We envisage that a differential screening scenario in a population screening program (such as the proposed DBT only for dense breasts) requires innovative studies and trials to determine how this could be evaluated in pragmatic implementation studies. Such an approach would also require consideration of ethical issues and stakeholder consultation.

## Electronic supplementary material

ESM 1 The incremental cost-effectiveness results at different costs of DBT (DOCX 20 kb)

## Abbreviations

CDR: Cancer detection rate
CHEERS: Consolidated Health Economic Evaluation Reporting Standards
DBT: Digital breast tomosynthesis
DM: Digital mammography
ICER: Incremental cost-effectiveness ratio
LYG: Life year gained
SE: Standard error
SiMRiSc: The simulation model on radiation risk and breast cancer screening
