# Supplementary material for: The cost-effectiveness of digital breast tomosynthesis in a population breast cancer screening program
Source: Eur Radiol. 2020 May 7;30(10):5437–45. doi: 10.1007/s00330-020-06812-x (PMC7476964; doi:10.1007/s00330-020-06812-x)
Supplement: Supplementary file 1 — The incremental cost-effectiveness results at different costs of DBT (DOCX 20 kb) [file 330_2020_6812_MOESM1_ESM.docx]

| The incremental cost-effectiveness results at different costs of DBT | | | | | |  |  |  |
| --- | --- | --- | --- | --- | --- | --- | --- | --- |
|  |  |  |  |  |  |  |  |  |
| Scenario 1: DBT for dense breasts only(at a DBT cost of €96) | | | |  | Scenario 1: DBT for dense breasts only(at a DBT cost of €80) | | | |
| Sensitivity(%) | ICER^1^(SD) | ICER^2^(SD) | ICER(SD) |  | Sensitivity(%) | ICER^1^(SD) | ICER^2^(SD) | ICER(SD) |
| **65** | **--** | **--** | **--** |  | **65** | **--** | **--** | **--** |
| **70** | **--** | **--** | **--** |  | **70** | **--** | **--** | **--** |
| **75** | **180265(37441)** | **120208(27608)** | **124063(32407)** |  | **75** | **91076(18891)** | **60742(13933)** | **62712(16348)** |
| **80** | **41021(3811)** | **26325(2479)** | **25542(2460)** |  | **80** | **20768(1920)** | **13345(1251)** | **12894(1235)** |
| **85** | **24407(1644)** | **15800(1094)** | **15378(1108)** |  | **85** | **12390(829)** | **8039(553)** | **7758(556)** |
| **90** | **17254(835)** | **11175(558)** | **10826572** |  | **90** | **8779(425)** | **5704(284)** | **5456(290)** |
| **95** | **13228(477)** | **8540(314)** | **8203(316)** |  | **95** | **6749(244)** | **4375(161)** | **4132(161)** |
| **100** | **11034(349)** | **7112(227)** | **6785(227)** |  | **100** | **5639(178)** | **3653(117)** | **3412(116)** |
|  |  |  |  |  |  |  |  |  |
| Scenario 2: DBT for whole population(at a DBT cost of €96) | | | |  | Scenario 2: DBT for whole population(at a DBT cost of €80) | | | |
| Sensitivity(%) | ICER^1^(SD) | ICER^2^(SD) | ICER(SD) |  | Sensitivity(%) | ICER^1^(SD) | ICER^2^(SD) | ICER(SD) |
| **65** | **--** | **--** | **--** |  | **65** | **--** | **--** | **--** |
| **70** | **--** | **--** | **--** |  | **70** | **--** | **--** | **--** |
| **75** | **--** | **--** | **--** |  | **75** | **--** | **--** | **--** |
| **80** | **--** | **--** | **--** |  | **80** | **--** | **--** | **--** |
| **85** | **63675(4488)** | **41216(2980)** | **42236(3157)** |  | **85** | **32201(2265)** | **20857(1505)** | **21344(1594)** |
| **90** | **31333(1327)** | **20048(851)** | **20146(872)** |  | **90** | **15884(673)** | **10180(432)** | **10171(442)** |
| **95** | **20771(537)** | **13232(342)** | **13171(346)** |  | **95** | **10557(274)** | **6743(175)** | **6644(176)** |
| **100** | **16013(368)** | **10188(226)** | **10072(219)** |  | **100** | **8156(187)** | **5207(115)** | **5074(110)** |
|  |  |  |  |  |  |  |  |  |
| Scenario2-scenario 1(at a DBT cost of €96) | | |  |  | Scenario2-scenario 1(at a DBT cost of €80) | | |  |
| Sensitivity(%) | ICER^1^(SD) | ICER^2^(SD) | ICER(SD) |  | Sensitivity(%) | ICER^1^(SD) | ICER^2^(SD) | ICER(SD) |
| **65** | **--** | **--** | **--** |  | **65** | **--** | **--** | **--** |
| **70** | **--** | **--** | **--** |  | **70** | **--** | **--** | **--** |
| **75** | **--** | **--** | **--** |  | **75** | **--** | **--** | **--** |
| **80** | **--** | **--** | **--** |  | **80** | **--** | **--** | **--** |
| **85** | **--** | **--** | **--** |  | **85** | **--** | **--** | **--** |
| **90** | **61857(2555)** | **39169(1503)** | **40107(1443)** |  | **90** | **31286(1294)** | **19824(761)** | **20268(732)** |
| **95** | **31877(949)** | **20123(648)** | **20441(717)** |  | **95** | **16166(480)** | **10222(328)** | **10320(361)** |
| **100** | **22066(555)** | **13912(346)** | **14032(352)** |  | **100** | **11216(282)** | **7090(176)** | **7077(177)** |
|  |  |  |  |  |  |  |  |  |
| Note: |  |  |  |  |  |  |  |  |
| ICER= Incremental cost-effectiveness ratio; SD=Standard deviation; DBT=Digital breast tomosynthesis | | | | | | |  |  |
| ICER^1^: Disounted by international guileline(3% for both cost and LYG); ICER2: Disounted by Dutch guileline(4% for cost and 1.5% for LYG); ICER: Undiscounted | | | | | | | | |
